# Accuracy of an automatic analysis software to detect microvascular density parameters

**DOI:** 10.1186/2197-425X-3-S1-A415

**Published:** 2015-10-01

**Authors:** A Carsetti, S Pierantozzi, HD Aya, S Bazurro, A Donati, A Rhodes, M Cecconi

**Affiliations:** Department of Biomedical Sciences and Public Health, Università Politecnica delle Marche, Ancona, Italy; Department of Intensive Care Medicine, St George's University Hospitals NHS Foundation Trust, London, United Kingdom; St George's University of London, London, United Kingdom

## Introduction

Analysis of microvascular density parameters is time consuming and operator-dependent.^1^ This is the main limitation to use microvascular monitoring in clinical practice as a “point-of-care” tool. Recently, an automatic analysis software has been developed and could allow us to obtain results quickly.

## Objectives

The aim of this study was to assess the accuracy of microvascular density parameters (total vessel density (TVD), perfused vessel density (PVD) and proportion of perfused vessels (PPV)) obtained by the new automatic analysis software CytoCamTools 1.7.12 (CC) (Braedius, Amsterdam, The Netherlands) in comparison with Automated Vascular Analysis (AVA) software 3.2 (MicroVision Medical, Amsterdam, The Netherlands).

## Methods

Sublingual microcirculatory videos were obtained using an incidence dark field-imaging device (CytoCam, Braedius, Amsterdam, The Netherlands). Only videos with a high quality score^2^ were selected for the analysis. Each video was analysed using AVA 3.2 by two skilled operators and results were compared with the analysis obtained by the CytoCamTools 1.7.12 software. Bland-Altman analysis was used to look at the agreement between the automatic software and the operators.

## Results

**Figure 1:**
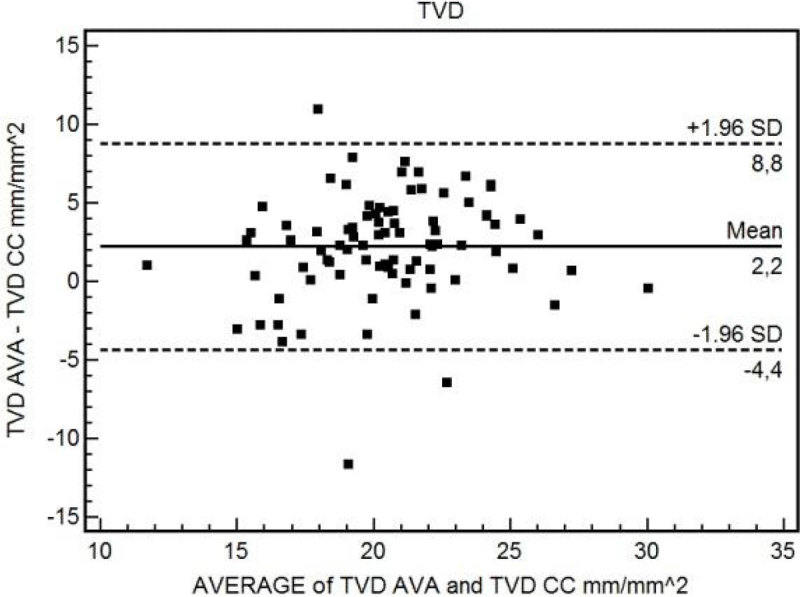
**Bland-Altman between TVD-AVA and TVD-CC.**

**Figure 2:**
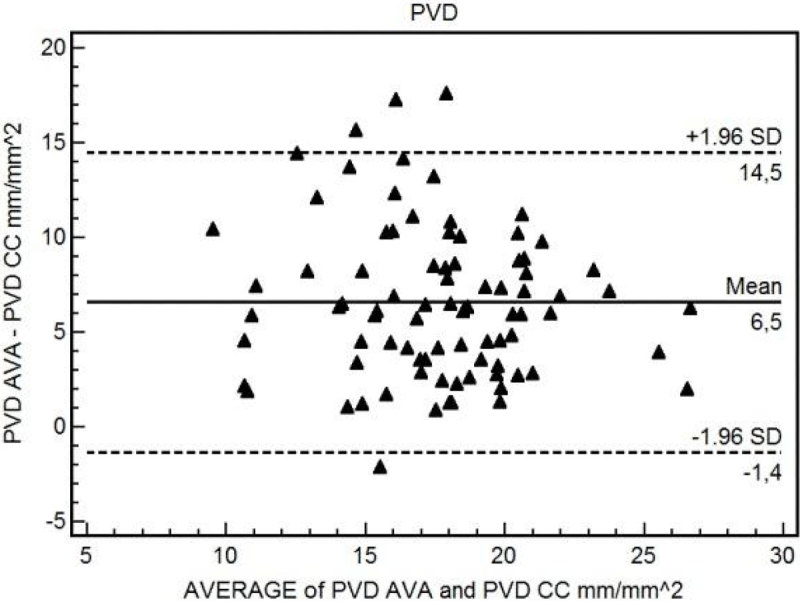
**Bland-Altman between PVD-AVA and PVD-CC.**

84 videos from 22 patients after cardiac surgery were analysed. The mean bias between TVD-CC and TVD-AVA was 2.17 mm/mm^2 (95% CI 1.44 to 2.90; p = 0.0001) with limits of agreement (LOA) of -4.41 (95% CI -5.66 to -3.16) and 8.76 (95% CI 7.50 to 10.01) mm/mm^2 (Figure 1). The mean bias between PVD-CC and PVD-AVA was 6.54 mm/mm^2 (95% CI 5.67 to 7.42; p < 0.0001) with LOA of -1.37 (95% CI -2.87 to 0.13) and 14.46 (95% CI 12.95 to 15.96) mm/mm^2 (Figure 2). A good correlation was found between the average of PPV-CC and PPV-AVA and the difference of the two measurements where ΔPPV = -1.85(mean PPV) +181 (R=-0.94, (95%CI -0.96 to -0.91; p < 0.0001).

## Conclusions

This study shows acceptable bias but wide limits of agreement for the comparison of TVD, PVD and PPV between the automated CC system and skilled operators. Further software improvements may be needed before real time point of care testing of the microcirculation can be used at the bedside.
